# Critical role of miR-10b in B-Raf^V600E^ dependent anchorage independent growth and invasion of melanoma cells

**DOI:** 10.1371/journal.pone.0204387

**Published:** 2019-04-17

**Authors:** Ila Datar, Gardiyawasam Kalpana, Jungmin Choi, Tupa Basuroy, Robert Trumbly, Sri Krishna Chaitanya Arudra, Michael D. McPhee, Ivana de la Serna, Kam C. Yeung

**Affiliations:** 1 Case Comprehensive Cancer Center, Case Western Reserve University, Cleveland, OH, United States of America; 2 Department of Cancer Biology, College of Medicine and Life Sciences, University of Toledo, Health Science Campus, Toledo, OH, United States of America; 3 Department of Genetics, Yale School of Medicine, New Haven, CT, United States of America; 4 Department of Pathology, Sanford Hospital, Bismarck, ND, United States of America; 5 ProMedica Cancer Institute, Toledo, OH, United States of America; Rutgers University, UNITED STATES

## Abstract

Recent high-throughput-sequencing of cancer genomes has identified oncogenic mutations in the *B-Raf* genetic locus as one of the critical events in melanomagenesis. *B-Raf* encodes a serine/threonine kinase that regulates the MAPK/ERK kinase (MEK) and extracellular signal-regulated kinase (ERK) protein kinase cascade. In normal cells, the activity of B-Raf is tightly regulated and is required for cell growth and survival. *B-Raf* gain-of-function mutations in melanoma frequently lead to unrestrained growth, enhanced cell invasion and increased viability of cancer cells. Although it is clear that the invasive phenotypes of *B-Raf* mutated melanoma cells are stringently dependent on B-Raf-MEK-ERK activation, the downstream effector targets that are required for oncogenic B-Raf-mediated melanomagenesis are not well defined. miRNAs have regulatory functions towards the expression of genes that are important in carcinogenesis. We observed that miR-10b expression correlates with the presence of the oncogenic *B-Raf* (*B-Raf*^*V600E*^) mutation in melanoma cells. While expression of miR-10b enhances anchorage-independent growth of *B-Raf* wild-type melanoma cells, miR-10b silencing decreases *B-Raf*^*V600E*^ cancer cell invasion *in vitro*. Importantly, the expression of miR-10b is required for B-Raf^V600E^-mediated anchorage independent growth and invasion of melanoma cells *in vitro*. Taken together our results suggest that miR-10b is an important mediator of oncogenic B-Raf^V600E^ activity in melanoma.

## Introduction

Melanoma is the most aggressive of all the skin cancers. B-Raf is a serine/threonine protein kinase that activates the MEK/ERK-signaling pathway. About 25–70% of malignant melanomas harbor gain-of-function mutations in the oncogene *B-Raf* [1]. Among several B-Raf gain-of-function mutations, B-Raf^V600E^ is the most common mutation and accounts for nearly 80% of them [1]. Not only does B-Raf^V600E^ cause a sustained activation of ERK signaling pathway in melanoma, it is also critical for the malignant process and is one of the few identified driver mutations essential for melanoma proliferation and survival. The transformation of melanocytes to melanoma by B-Raf^V600E^ requires activation of the MEK-ERK kinases cascade with multiple downstream components [2–4]. The mechanism that integrates the diverse components into a coordinated response to the B-Raf^V600E^ mutation remains undefined.

MicroRNAs (miRNAs) are small, non-protein coding RNA molecules and they regulate gene expression through a combination of translational repression and mRNA destabilization [5]. Each miRNA targets ~200 mRNA molecules [6]. Because of their pleiotropic potentials, miRNAs are attractive candidates as master regulators of the B-Raf^V600E^ oncogenic transformation program. In this study, we identified for the first time, significant positive correlation between B-Raf^V600E^ mutation and microRNA-10b (miR-10b) expression. Furthermore, we show that miR-10b is a novel downstream effector of B-Raf^V600E^ and that B-Raf^V600E^ plays a causal role in the induction of miR-10b in melanoma cell lines. Our results suggested that B-Raf ^V600E^ increased miR-10b expression by increasing the expression levels of helix-loop-helix transcription factor Twist1. We also show that miR-10b induced by B-Raf^V600E^ is able to increase invasive capacity and anchorage independent growth of melanoma cells.

## Materials and methods

### Cell culture

All cell culture media were from HyClone. Fetal bovine serum (FBS) was from Atlanta Biologicals, and newborn calf serum was from Lonza. 10 cm^2^ and 6 cm^2^ cell culture plates were from Sarstedt. All the melanoma cell lines were cultured in Dulbecco’s modified Eagle’s medium with 10% FBS and 1% penicillin streptomycin and were grown in a humidified tissue culture incubator at 37°C in 5% CO2. Mel 505 [7], PMWK [8], sk-mel-28 [9], sk-mel-24 [7], VMM39 [7] and MEL 224 [7] cells were kindly provided by Dr. J. Shields (University of North Carolina, Chapel Hill). YUHEF [10] and YUROB [10] cells were a kind gift from Dr. R. Halaban (Yale University, Connecticut). sk-mel-197 [11] cells were received from Memorial Sloan Kettering Cancer Center. M249 [12] cells were a kind gift from Dr. A. Ribas (UCLA).

### Chemicals and reagents

PLX4032 (Vemurafenib) and 4-OHT (4-hydroxy tamoxifen) were purchased from Selleck chemicals and Sigma, respectively. ERK (1:1000), phospho ERK (1:1000), B-Raf (1:1000) primary antibodies and horseradish peroxidase secondary antibodies were from Santa Cruz Biotechnology, Twist1 (1:500) antibody was from R&D systems, and tubulin (1:1000) antibody was from Sigma.

### Ectopic expression and depletion of miR-10b

Mammalian expression vectors, MDH1-PGK-GFP 2.0-miR-10b and pBABE-puro-miR-10b sponge, were purchased from Addgene (Plasmids were deposited by Weinberg lab) [13]. Melanoma cells were stably transduced with viral particles expressing miR-10b or miR-10 sponge as described previously [14].

### *In vitro* Matrigel invasion assay

The polycarbonate membrane (8μ pore size) of FluoroBlok cell culture inserts (BD Biosciences) was coated with 60 uL of Matrigel (1:26 in serum-free medium) (BD Biosciences) and incubated at 37°C for 2–3 hours. 1x10^4^ of sk-mel-28 or M249 cells were plated on these inserts. 700mL of chemo-attractive medium (Dulbecco’s Modified Eagle’s medium, 1% P/S and 10% FBS) was added to the lower chambers (24-well BD Falcon TC companion plate). After 24 hours of incubation, the insert bottoms were dipped in 1X PBS and stained in Calcein AM reconstituted in DMSO to 1mg/mL- 1ml in 700 ml 1X PBS (BD Biosciences). Digital images were captured on EVOS inverted microscope along with manual cell count and fluorescence reading (485-538nM).

### ChIP assay

Chromatin Immunoprecipitations (ChIPs) were performed as described previously [15]. Briefly, cells were crosslinked with 1% formaldehyde for 10 minutes at room temperature. Chromatin was sheared by sonication and immunoprecipitated overnight with control rabbit IgG or an antibody to Twist (Rabbit polyclonal Anti-Twist, Santa Cruz (H-81), sc15393). Following reversal of cross-links, the DNA was purified by proteinase K digestion followed by phenol-chloroform extraction and ethanol precipitation. The purified DNA was subjected to quantitative real-time PCR using SYBR Green Master Mix (Qiagen) with an Applied Biosystems Prism 7500 PCR system and analyzed with the SDS software. Primers for Ebox1 or Ebox2 were described previously [13].

### RNA extraction and TaqMan miRNA quantitative real-time PCR assay

RNA extraction was carried out using Qiazol reagent (Qiagen). TaqMan miRNA qRT-PCR was performed according to manufacturer’s instructions (TaqMan). RNU6 or RNU44 were used as the internal control. Briefly, in the reverse transcription (RT) step, cDNA was reverse transcribed from total RNA samples using specific looped miRNA primers from the TaqMan miRNA assays and reagents from the TaqMan. In the PCR step, PCR products were amplified from cDNA samples using the TaqMan miRNA assay together with the TaqMan universal PCR master mix.

### Soft agar assay for anchorage independence

1mL layer of 1:1 mixture of 1.2% agar and 2X medium (to give a final 0.6% agar concentration) was evenly spread on 35mm plates. It was allowed to set for 30 minutes at RT. Cells were counted and plated in a mixture of 1.2% agar and 2X medium (1:4) to give a final agar concentration of 0.3%. 3x10^3^ of sk-mel-28 cells or 1x10^4^ of Mel 505 cells or 1x10^4^ of sk-mel-197 cells were plated. Number of colonies formed were stained with MTT and counted under bright field microscope at 4X objective. Cells were plated in triplicates and four fields were counted per 35 mm plate.

### TCGA data analysis

The publicly available TCGA data were directly downloaded from the TCGA Data Portal at https://tcga-data.nci.nih.gov/. TCGA melanoma cases (TCGA-SKCM) annotated for B-Raf WT or V600E mutation were extracted from the GDC portal (https://portal.gdc.cancer.gov). Among those, 407 samples were profiled for both mRNA expression and miRNA expression. Fragments per Kilobase per Million mapped reads (FPKM) from the RNA-sequencing data and reads per million miRNA mapped from the miRNA data were used as normalized read counts. Pearson correlation coefficients and P-values for miR-10b expression with Twist1 and MYC expression were calculated, respectively.

### Western blot analysis

Cell extracts were prepared and western blotting was carried out. Briefly, cells were lysed with 20 mM Tris, pH 7.4, 150mM NaCl, 2mM EDTA, and 1% Triton X-100. Samples (10–50 μg protein) were separated by sodium dodecyl sulfate-polyacrylamide electrophoresis and then electrophoretically transferred from the gel to polyvinylidene fluoride membranes (Millipore). All the primary antibodies were diluted in phosphate buffered saline, pH 7.4, 0.2% Tween-20, 5% bovine serum albumin and 0.002% sodium azide. Following three washes, blots were incubated with the appropriate horseradish peroxidase-conjugated secondary antibody for 1 h at room temperature. Proteins were visualized with enhanced chemiluminescence with the BioRad ChemiDoc EQ system.

### Statistical analysis

All experiments were performed in triplicates. All the statistical analyses were performed using the two-tailed student’s t-test (GraphPad Prism software).

## Results and discussion

**Fig 1:**
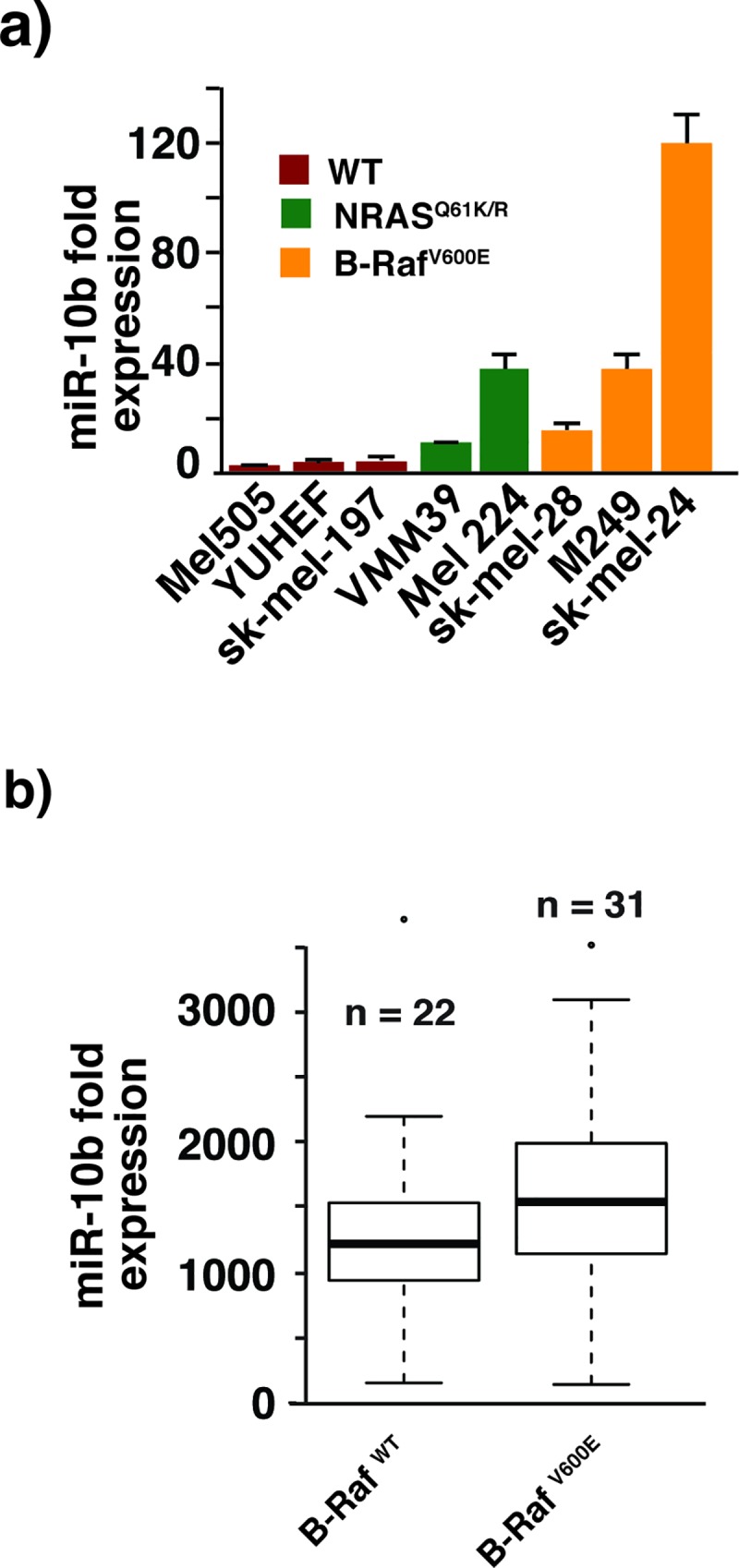
miR-10b expression positively correlates with B-Raf^V600E^ mutation status in multiple cell lines. (a) TaqMan reverse transcriptase PCR shows a strong positive correlation of miR-10b expression with B-Raf^V600E^ or N-Ras activating mutations in a panel of melanoma cell lines. The experiment was repeated 3X with similar results. (b) A boxplot graph compares miR-10b expression levels in 22 wild-type and 31 B-Raf ^V600E^ mutant melanoma cell lines. The mean value for the wild-type cells was 1254.7, and for the mutant cells 1627.4. The p-value for a one-tailed t-test was 0.0422. The data are from GSE89438 [18]. The expression values for miR-10b were downloaded as part of the GSE89438_series_matrix file from NCBI GEO. The miR-10b values were imported into the R programming environment, and the boxplot function was applied to the values for wild-type and B-Raf^V600E^ mutant samples.

We previously reported that the expression of several miRNAs was altered upon introduction of B-Raf^V600E^ in primary melanocytes [16]. Among the miRNAs whose expression levels were affected by B-Raf^V600E^, we chose miR-10b for further study because its expression positively correlates with V600E mutation in B-Raf in established melanoma cell lines (Fig 1), clinical melanoma samples (S1 Fig), and because it is involved in several other malignancies [17].

**Fig 2:**
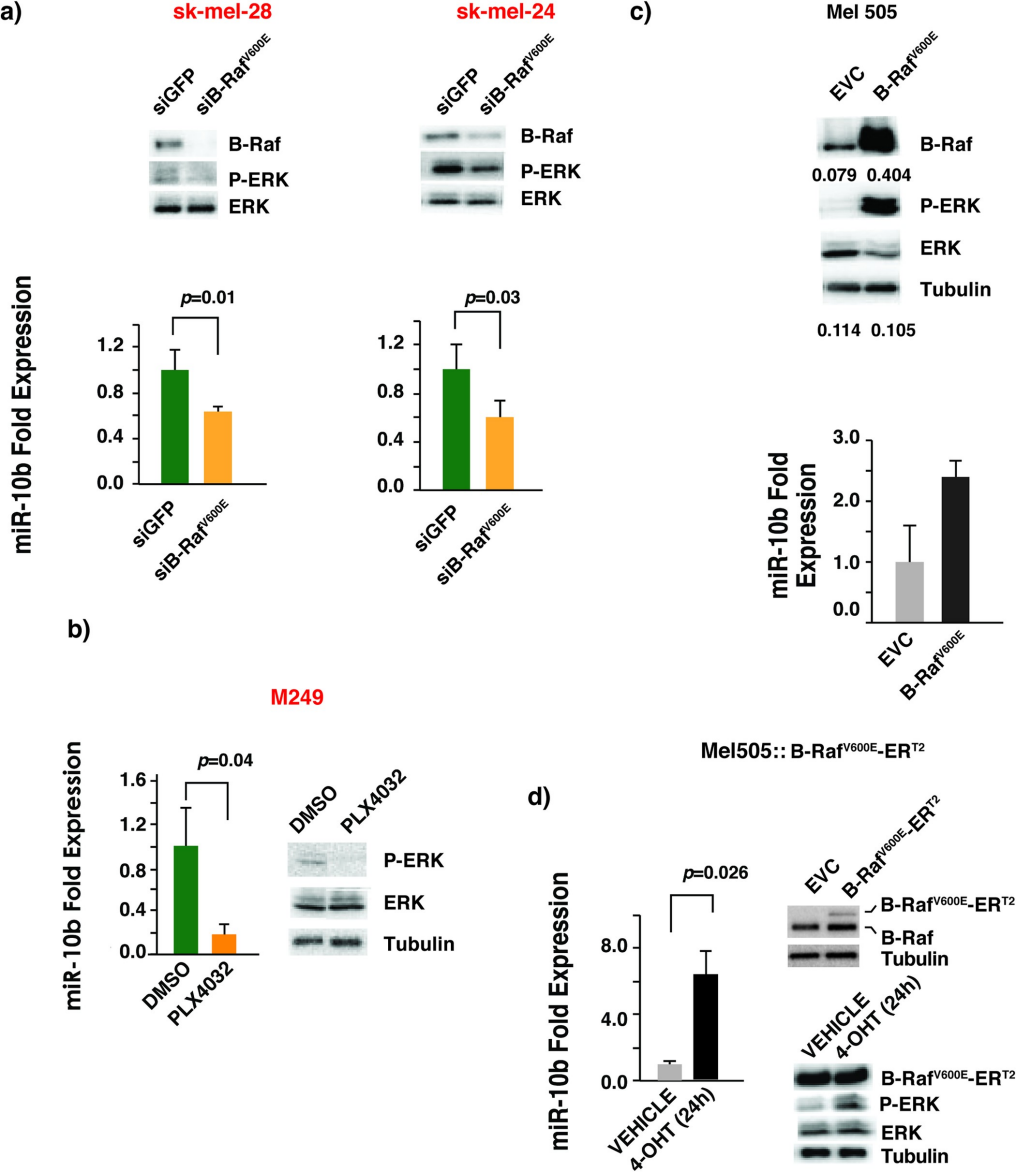
B-Raf^V600E^ plays a causal role in the induction of miR-10b. (a) Lower panel shows downregulation of miR-10b expression upon stable knock-down of B-Raf^V600E^ in melanoma cell lines sk-mel-24 and sk-mel-28 with B-Raf^V600E^-specific siRNA as determined by TaqMan qRT-PCR. Upper panel: Western blots of lysates from cells expressing siB-Raf^V600E^ or siGFP siRNA. (b) TaqMan qRT-PCR shows downregulation of miR-10b upon inhibition of the B-Raf^V600E^ in melanoma cell line M249 by PLX4032. Cells were treated at 1μM concentration of PLX4032 (Vemurafenib) or DMSO for a period of 12 hours. (c) Increase in the expression of miR-10b upon ectopic expression of B-Raf^V600E^. Cells were stably transduced with B-Raf^V600E^ - or empty vector control (EVC)-expressing retroviruses. Relative levels of B-Raf and tubulin are indicated. (d) Potent induction of miR-10b in B-Raf wild type Mel 505 cell line upon activation of the transduced B-Raf^V600E^-ER^T2^ following 24 hours 4HT (4-hydroxy tamoxifen) treatment at 1μM concentration as determined by TaqMan qRT-PCR (left panel). Cells were stably transduced with B-Raf^V600E^-ER^T2^-expressing retrovirus. The expression levels of B-Raf^V600E^-ER^T2^ in transduced cells were measured with Western blotting with specific B-Raf antibodies (Upper right panel). The effects of 4HT activated B-Raf^V600E^-ER^T2^ in transduced Mel505 cells on ERK phosphorylation were monitored with Western blotting with indicated specific antibodies (Lower right panel).

To investigate if B-Raf^V600E^ plays a causal role in regulating miR-10b expression in melanoma cell lines we undertook a loss-of-function approach. We stably knocked down B-Raf^V600E^ expression by siRNA in two different B-Raf^V600E^ melanoma cell lines. As expected, B-Raf protein expression significantly decreased in B-Raf^V600E^ knockdown cells with a concomitant decrease in phosphorylation of ERK1/2. In both studied cell lines, we found that miR-10b expression was significantly down-regulated upon B-Raf^V600E^ knock down (Fig 2A). A decrease in miR-10b expression was also observed in M249, which are B-Raf^V600E^ positive melanoma cells when B-Raf^V600E^ activity was down-regulated by vemurafenib (PLX4032), a pharmacological inhibitor of B-Raf^V600E^ (Fig 2B).

We reasoned that if the effect of knocking down of B-Raf^V600E^ expression on miR-10b is physiological we should observe an opposite effect with over-expression of the oncogenic B-Raf^V600E^ in melanoma cell lines carrying wild-type B-Raf. Indeed, we found that the expression of miR-10b was significantly upregulated in cells ectopically expressing B-Raf^V600E^ as compared to empty vector control (Fig 2C). The drawback of over-expression of an oncogene is that the cells might get adapted to its sustained expression and this can lead to non-specific effects that may not be attributable to the expression of the oncogene alone. As a complementary approach, we used an estrogen receptor fusion system where B-Raf^V600E^ was rendered functionally hormone-dependent by fusion with synthetic steroid 4-hydroxytamoxifen (4-OHT)-binding domain of mutated estrogen receptor (ER^T2^) [19]. As expected, upon treating the cells with 4-OHT for 24 hours, we observed potent induction of ERK dual phosphorylation at Thr^202^ and Tyr^204^ indicating that the kinase activity of B-Raf^V600E^ was temporally turned on by 4-OHT treatment (Fig 2D, lower right panel). Interestingly, transient activation of B-Raf^V600E^ is better at activating miR-10b expression than sustained expression of the constitutively active kinase (Compare Fig 2C and 2D). Taken together, our results suggest that the expression of miR-10b in melanoma is B-Raf^V600E^ dependent.

**Fig 3:**
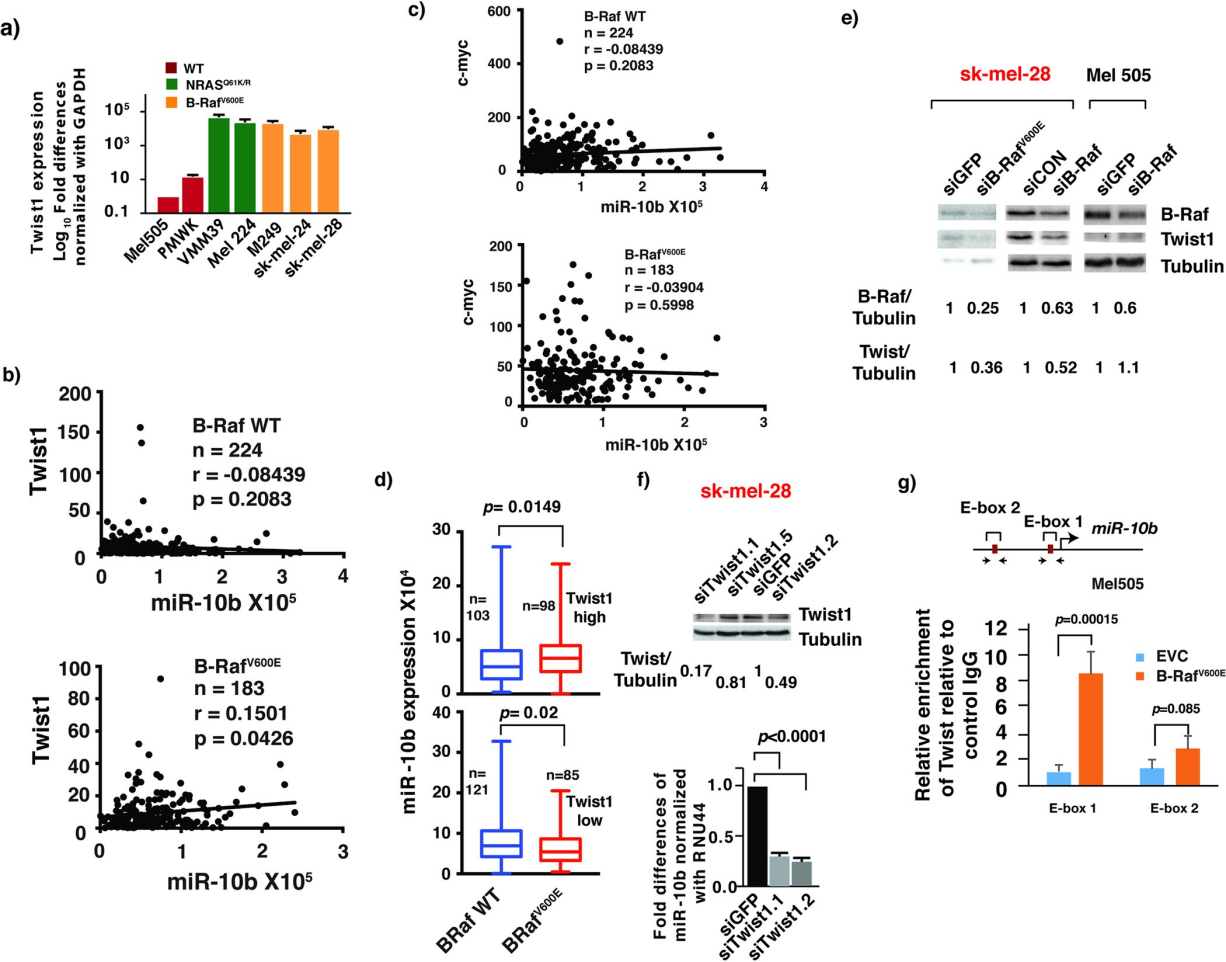
The B-Raf^V600E^-Twist axis regulates miR-10b expression in melanoma. (a) Twist1 expression positively correlates with B-Raf^V600E^ mutation status in multiple cell lines. qRT- PCR shows a strong positive correlation of Twist1 expression with B-Raf^V600E^ or N-Ras activating mutations in a panel of melanoma cell lines. (b-c) miR-10b displays significant correlation with Twist1 mRNA expression in B-Raf^V600E^ mutant melanoma. Scatter plots showing correlation of miR-10b expression with (b) Twist1 mRNA expression and (c) Myc mRNA expression in B-Raf^WT^ and B-Raf^V600E^ samples from 407 TCGA-SKCM samples. r denotes Pearson correlation coefficient. p refers to a Pearson correlation P-value. (d) Upper panel: Box plots display miR-10b expression distribution in B-Raf^WT^ and B-Raf^V600E^ from 201 TCGA-SKCM samples where Twist1 mRNA expression was high. The median expression value for the wild-type samples was 50,343, and for the mutant was 66,002. The *p*-value for a Mann-Whitney test was 0.0149. Lower panel: Box plots display miR-10b expression distribution in BRaf^WT^ and BRaf^V600E^ from 206 TCGA-SKCM samples where Twist1 mRNA expression was low. The median expression value for the wild-type samples was 69,160, and for the mutant was 54,373. The *p*-value for a Mann-Whitney test was 0.02. (e) Protein expression of B-Raf and Twist1 in sk-mel-28 or Mel505 cell lines expressing siRNAs against B-Raf or control siRNAs (siGFP or siCON). Relative levels of B-Raf or Twist1 are indicated. (f) Upper panel, relative Twist1 mRNA expression in sk-mel-24 cells expressing three different Twist1 specific siRNAs or siGFP control as assessed by qRT-PCR. Lower panel, relative mR-10b expression in the indicated siRNA expressing sk-mel-24 cells as assessed by TaqMan qRT-PCR. (g) ChIP analysis on M505 cells with or without the ectopic expression of B-Raf^V600E^. The putative E-boxes in miR-10b promoter are shown as red boxes. The locations of primers used for ChIP assays are marked by black arrows (amplicons). ChIP analysis was conducted using specific antibodies that recognize human Twist1, as well as control IgG.

In humans, the *miR-10b* gene resides upstream from the developmental gene *Hoxd4*. It has been shown that the basic helix-loop-helix transcription factor Twist positively regulates miR-10b expression in breast cancer cells by directly binding to the proximal E-box present in the *miR-10b* gene promoter. The expression levels of Twist are often deregulated in a vast majority of melanoma tumors and cell lines [20, 21]. Furthermore, the expression of Twist was shown to be regulated at the transcription level by B-Raf^V600E^ mutation in human melanoma cells [22], raising the possibility that B-Raf^V600E^ may increase miR-10b expression by activating Twist. Indeed, hyper activation of B-Raf signaling pathway increases Twist1 transcript in established melanoma cell lines (Fig 3A). In addition, we also observed significant correlation of miR-10b and Twist1 expression in B-Raf^V600E^ mutated but not B-Raf wild-type melanoma clinical samples (Fig 3B and Table 1). The observed correlation is specific as no significant correlation was observed between another E-box transcription factor c-myc and miR-10b (Fig 3C and Table 2). Importantly, knocking down of B-Raf^V600E^ diminished Twist1 expression while the expression of Twist was important for miR-10b synthesis (Fig 3D–3F, S2 Fig). It is of interest to note that knocking down of wild type B-Raf in Mel505 cells had no noticeable effect on the expression levels of Twist1 (Fig 3E, right panel). Finally, Twist1 may have a direct effect on miR-10b expression as the promoter occupancy of *miR-10b* by Twist1 was elevated upon exogenous expression of B-Raf^V600E^ in B-Raf wild-type Mel505 melanoma cell line (Fig 3G). Our results therefore suggested that the activation miR-10b expression by B-Raf^V600E^ required the presence of Twist1 in melanoma. It is possible that B-Raf gain-of-function mutation is necessary but not sufficient for miR-10b expression in melanoma. Indeed, we did not observe significant correlation of B-Raf mutation status and miR-10b expression level in melanoma TCGA datasets before the clinical samples were stratified into Twist1 positive and negative groups (Fig 3D).

**Table 1:** Pearson correlation statistics between Twist1 and miR-10b expression in B-Raf^WT^ and B-Raf^V600E^.

Twist1 vs miR-10b (B-Raf^WT^)		Twist1 vs miR-10b (B-Raf^V600E^)	
Pearson r		Pearson r	
r	-0.08439	r	0.1501
95% confidence interval	-0.2131 to 0.04721	95% confidence interval	0.005107 to 0.2888
R squared	0.007122	R squared	0.02252
P value		P value	
P (two-tailed)	0.2083	P (two-tailed)	0.0426
P value summary	ns	P value summary	*
Significant? (α = 0.05)	no	Significant? (α = 0.05)	yes
Number of XY Pair	224	Number of XY Pair	183

r denotes parametric Pearson correlation coefficient and r^2^ (R squared) indicates the coefficient of determination.

* indicates the significance of the P value. Two-tailed P values were calculated for Pearson correlation coefficients.

**Table 2:** Pearson correlation statistics between Myc and miR-10b expression in B-Raf^WT^ and B-Raf^V600E^.

c-Myc vs miR-10b (B-Raf^WT^)		c-Myc vs miR-10b (B-Raf^V600E^)	
Pearson r		Pearson r	
r	0.08854	r	-0.03904
95% confidence interval	-0.04304 to 0.2171	95% confidence interval	-0.1831 to 0.1066
R squared	0.007839	R squared	0.001524
P value		P value	
P (two-tailed)	0.1867	P (two-tailed)	0.5998
P value summary	ns	P value summary	ns
Significant? (α = 0.05)	no	Significant? (α = 0.05)	no
Number of XY Pair	224	Number of XY Pair	183

r denotes parametric Pearson correlation coefficient and r^2^ (R squared) indicates the coefficient of determination. Two-tailed P values were calculated for Pearson correlation coefficients.

**Fig 4:**
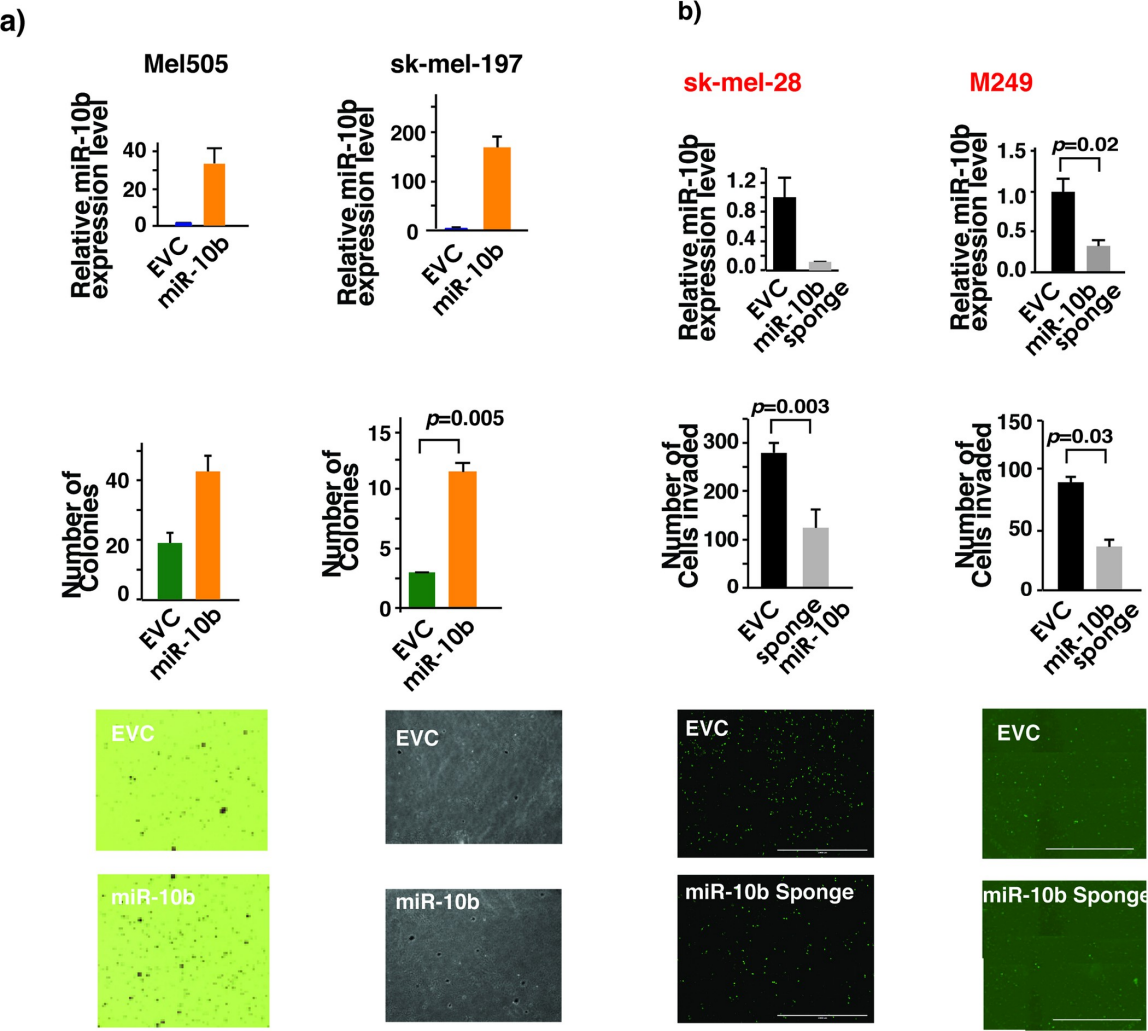
miR-10b confers anchorage-independence and increases invasive capacity of melanoma cell lines. (a) Middle two panels show increase in the number of colonies formed in soft-agar upon ectopic expression of miR-10b in Mel 505 and sk-mel-197 melanoma cell lines expressing WT B-Raf. Lower panels are pictures of cells shown in the middle panel photographed at X40 after staining with MTT. Upper panel shows miR-10b fold expression upon ectopic expression of miR-10b. Student’s t-test was performed for statistical analysis, which shows a significant value of 0.002 for Mel 505 and 0.005 for sk-mel-197 cell lines. (b) Middle panels show decrease in number of invading cells upon miR-10b depletion by specific miR-10b sponge in sk-mel-28 and M249 cells. Lower panels show representative fields of Matrigel membranes with invaded cells stained fluorescent green with Calcein AM. Student’s t-test was performed and a significant difference was found with a p-value of 0.0001. Upper panel shows expression levels of miR-10b in miR-10b sponge expressed sk-mel-28 and M249 cells as determined by TaqMan qRT-PCR.

**Fig 5:**
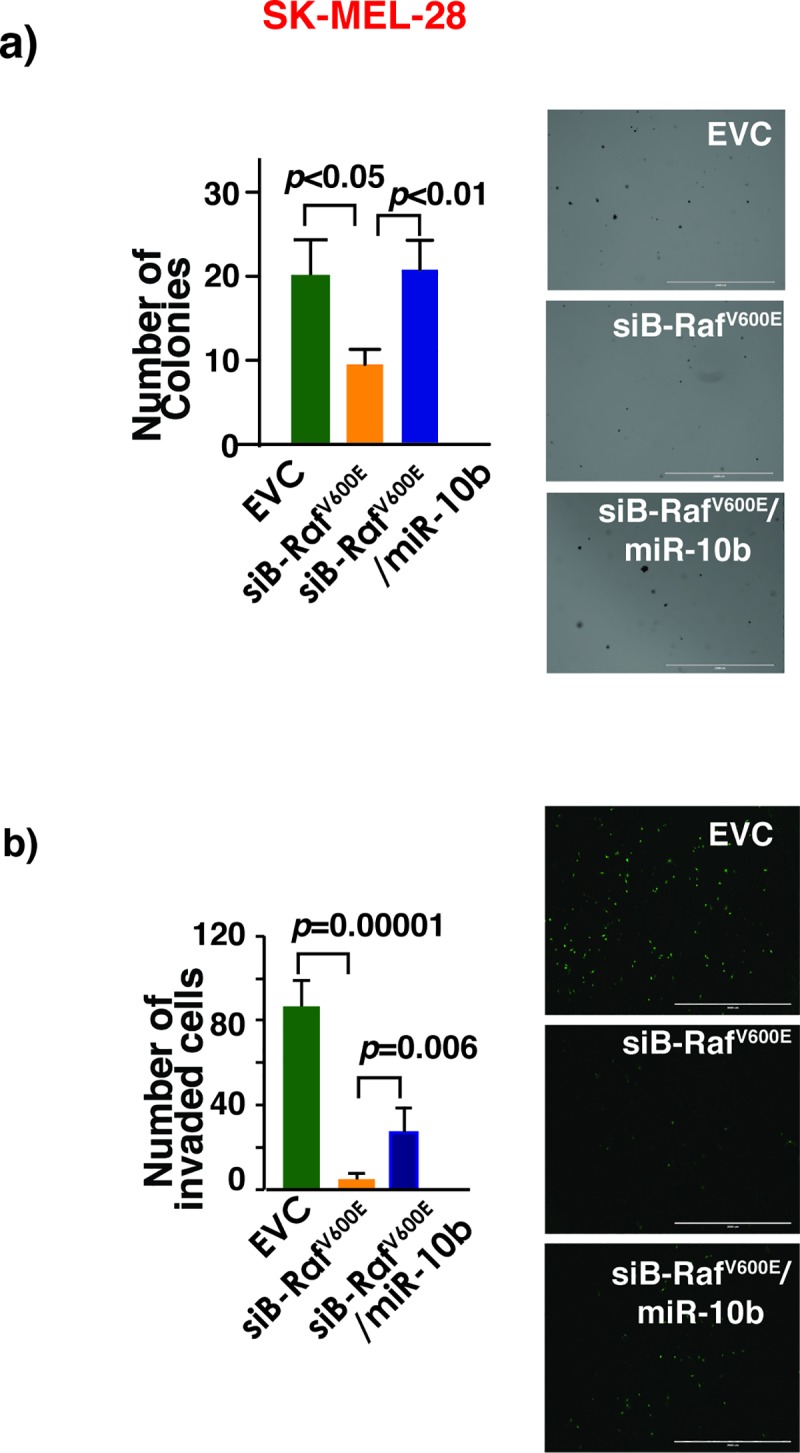
B-Raf^V600E^ melanoma cells acquire cancer phenotypes partly by increasing the expression levels of miR-10b. (a) Upper panel: B-Raf^V600E^ knockdown decreases number of colonies in soft agar in sk-mel-28 cell line. Restoration of miR-10b expression reversed this effect. This experiment was performed at least twice with triplicates each time. ANOVA was performed followed by Bonferroni’s multiple comparison test where EVC vs siB-Raf^V600E^ and B-Raf^V600E^ vs B-Raf^V600E^ /miR-10b were found to be significant with a p-value <0.05. EVC-empty vector control. (b) Knockdown of B-Raf^V600E^ decreases melanoma cell invasion while restoration of miR-10b reverses this effect. Right panel shows a representative field of Matrigel membrane with invading cells staining fluorescent green with Calcein AM. One-way ANOVA was performed with p-value<0.05.

Studies with cancer cell transplantation and autochthonous cancer mouse models have demonstrated the causal role of miR-10b in breast cancer initiation, growth, progression and metastasis [23–25]. However, the causality of loss- or gain-of-function of miR-10b in other cancers including melanoma is not known. Anchorage-independent growth and increased invasive capacity are two well-characterized hallmarks of cancer. Since Twist-miR-10b axis has a demonstrated role in anchorage-independent growth [26], miR-10b may confer these important cancerous phenotypes on melanoma cells. Indeed, in low-miR-10b-expressing wild-type *B-Raf* melanoma cells Mel505, ectopic expression of miR-10b was sufficient to increase anchorage-independent growth as measured in soft agar (Fig 4A). This effect was not cell type specific as a similar effect was observed with other wild-type *B-Raf* melanoma cells sk-mel-197. Similarly, depletion of miR-10b in high-miR-10b-expressing *B-Raf*^*V600E*^ melanoma cells sk-mel-28 and M249 decreased their invasiveness (Fig 4B). B-Raf^V600E^ is an oncoprotein and its presence is essential for anchorage-independent growth and invasive capacity of *B-Raf*^*V600E*^ melanoma cells. B-Raf^V600E^ induces miR-10b expression in melanoma cell lines. Therefore, *B-Raf*^*V600E*^ melanoma cells may acquire cancer phenotypes partly by increasing the expression levels of miR-10b through increasing the expression of the transcription factor Twist1. Consistent with this line of thinking, as shown in Fig 5A and 5B, ectopic miR-10b expression significantly rescued the decrease in anchorage independent growth and invasiveness due to the loss of B-Raf^V600E^ in sk-mel-28 cells.

The mechanisms of how augmented miR-10b expression in B-Raf^V600E^ melanoma cells enhances their anchorage independent growth and invasiveness is currently not known. miRNAs function mainly by silencing expression of target genes by binding to specific sites of the targeted gene mRNAs. Studies of genetic deletion of miR-10b in MMTV-PyMT mice identified *Tbx5*, *Pten*, and *HoxD10* as key miR-10b targets [27]. While *Tbx5* and *Pten* are well-characterized tumor suppressor genes, *HoxD10* is a proven metastasis suppressor gene. To grow continuously after being detached from extracellular matrix, cancer cells develop resistance to a specific form of caspase-mediated cell death known as anoikis by upregulating the survival pathways. PI3K pathway is a major survival pathway cancer cells employ to overcome anoikis to achieve anchorage-independent growth [28]. Of the three target genes of miR-10b, *Pten* has been shown to play a pivotal role in dampening the PI3K pathway. It is therefore possible that miR10b enhances anchorage independent growth in melanoma by decreasing the expression of Pten. Both Tbx2 and HoxD10 are transcription factors that can potentially regulate a multitude of genes. It has been shown that HoxD10 represses the expression of metastasis gene *RhoC* in breast cancer cells and that miR-10b increases cancer cells invasion by repressing the repressor of *RhoC* expression. *RhoC* is a well-studied metastasis gene in melanoma and it is possible that B-Raf^V600E^ utilizes the same miR-10b-HoxD10-RhoC axis to drive cancer cells invasion. Likewise, Tbx2 can also play an equally important role in mediating the different effects on melanomagenesis resulting from B-Raf mutation.

In 2010, an orally available inhibitor of B-Raf^V600E^ increased the median progression-free survival of patients with malignant melanoma harboring this mutation, to more than 7 months [29]. However, patients develop resistance to this drug due to several mechanisms including receptor tyrosine kinase (RTK) or N-Ras upregulation among many others [30]. Recently, combinatorial therapy utilizing B-Raf and MEK inhibition delayed the emergence of resistance while progression-free survival remained more or less similar to B-Raf inhibition alone [31]. However, relapse due to drug-resistance is commonplace and warrants a treatment strategy based on new downstream molecular targets of the mutant B-Raf protein. The expression of miR-10b is often deregulated in cancers including melanoma and its expression was positively associated with worse patient survival [24]. Here we identify miR-10b as a novel molecular target downstream of mutant B-Raf protein raising the possibility that targeting miR-10b and/or its effectors may represent a rational alternative treatment for relapsed patient with B-Raf^V600E^ melanoma.

## Supporting information

S1 Text miRNA *in situ* hybridization. (DOCX)

S1 Fig Expression levels of miR-10b in clinical melanoma samples as determined by ISH with labeled specific miRNA probe. (n = 4). The B-Raf^V600E^ mutation was determined by IHC staining with B-Raf^V600E^ specific Ab. Lesions were demarcated from normal tissues with white dotted line.
(TIF)

S2 Fig Expression levels of miR-10b in Twist1 knockdown B-Raf^V600E^ sk-mel-24 cells as determined by TaqMan qRT-PCR. Upper panel shows the relative Twist1 mRNA expression in sk-mel-24 cells expressing two different Twist1 specific siRNAs or siGFP control as assessed by qRT-PCR. Lower panel shows the relative mR-10b expression in the indicated siRNA expressing sk-mel-24 cells as assessed by TaqMan qRT-PCR.
(TIF)
