# Supplementary material for: Critical role of miR-10b in B-RafV600E dependent anchorage independent growth and invasion of melanoma cells
Source: PLoS One. 2019 Apr 17;14(4):e0204387. doi: 10.1371/journal.pone.0204387 (PMC6469749; doi:10.1371/journal.pone.0204387)
Supplement: S1 Text — (DOCX) [file pone.0204387.s001.docx]

**S1 Methods**

**miRNA *in situ* hybridization (ISH)**

6 µm thick sections were cut in RNase-free conditions and the sections were floated in DEPC-treated water in a water bath at 45^0^C. The in situ hybridization was performed according to manufacturer’s instructions (Exiqon miRNA-LNA hybridization protocol). Briefly, all the glassware and slides were first autoclaved and then heat-treated at 180^0^C for 8-10 hours. To make RNase-free water, milliQ water was treated with DEPC and stirred overnight followed by autoclaving. All the buffers and reagents were prepared in DEPC treated water, in heat-treated bottles and autoclaved. The staining jars were

sprayed with RNase-zap spray and rinsed with RNase-free DEPC water. The sections

were deparaffinized, treated with proteinase-k at 37^0^C for 10 mins and dehydrated. This

was followed by hybridization of the double-digoxigenin(DIG) labeled miRNA-LNA

probe specific for miR-10b which served as positive control and scrambled

probe served as negative control at 53^0^C for 1 hour. The hybridization was followed by

stringent washes at 53^0^C, blocking, addition of anti-DIG-AP antibody, addition of

substrate NBT/BCIP and counterstaining with neutral red. The slides were then

dehydrated and mounted. They were allowed to dry overnight and brightfield images

were obtained using 20X and 40X magnification.

**Melanoma FFPE Blocks**

Archived and deidentified melanoma samples were obtained from Promedica Cancer Institute.They were sectioned and stained for BRaf^V600E^ using a mutation-specific antibody to determine their mutation status. The sections were then stained for miR-10b expression using the ISH protocol mentioned above.
