# Supplementary figures and images for: Critical role of miR-10b in B-RafV600E dependent anchorage independent growth and invasion of melanoma cells
Source: PLoS One. 2019 Apr 17;14(4):e0204387. doi: 10.1371/journal.pone.0204387 (PMC6469749; doi:10.1371/journal.pone.0204387)

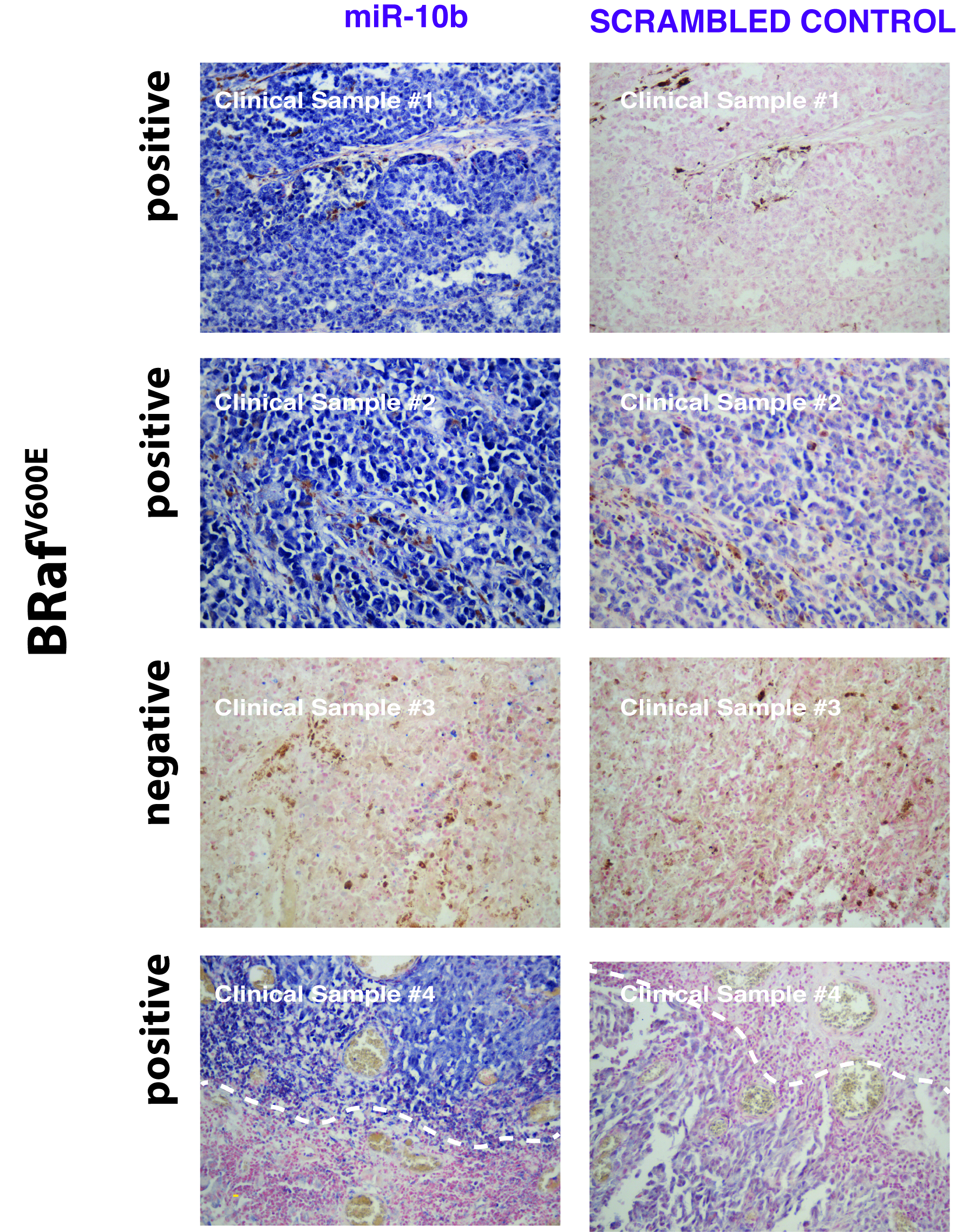

Supplement: S1 Fig — (n = 4). The B-RafV600E mutation was determined by IHC staining with B-RafV600E specific Ab. Lesions were demarcated from normal tissues with white dotted line. (TIF) [file pone.0204387.s002.tif]

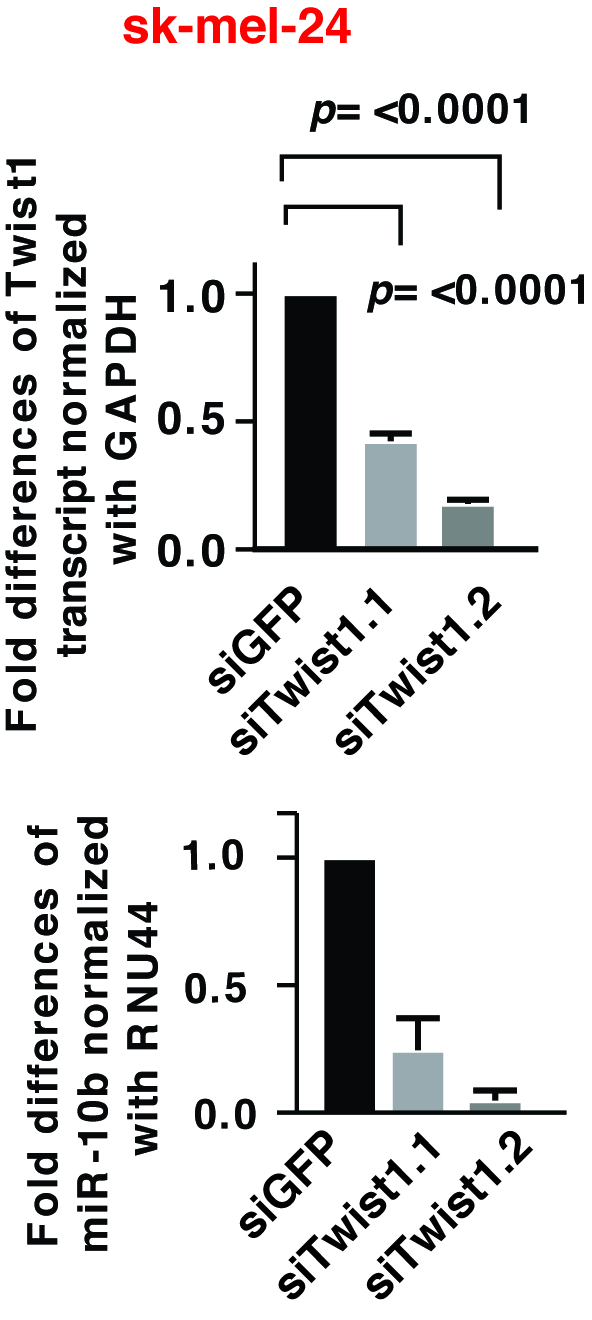

Supplement: S2 Fig — Upper panel shows the relative Twist1 mRNA expression in sk-mel-24 cells expressing two different Twist1 specific siRNAs or siGFP control as assessed by qRT-PCR. Lower panel shows the relative mR-10b expression in the indicated siRNA expressing sk-mel-24 cells as assessed by TaqMan qRT-PCR. (TIF) [file pone.0204387.s003.tif]
